# Intraspecific variations in leaf functional traits of *Cunninghamia lanceolata* provenances

**DOI:** 10.1186/s12870-023-04097-y

**Published:** 2023-02-13

**Authors:** Rui Xu, Shundan Cheng, Jing Zhou, Mulualem Tigabu, Xiangqing Ma, Ming Li

**Affiliations:** 1 College of Forestry, Fujian Agriculture and Forestry University, Fuzhou, 350002 China; 2 Southern Swedish Forest Research Center, Faculty of Forest Science, Swedish University of Agricultural Sciences, P.O. Box 190, 234 22 Lomma, Sweden; 3 Chinese Fir Engineering Technology Research Center of National Forestry and Grassland Administration, Fuzhou, 350002 Fujian China

**Keywords:** *Cunninghamia lanceolata*, Leaf dry mass, Specific life area, Nutrient content

## Abstract

**Background:**

Studies on intra-specific variability in leaf functional traits is important to evaluate adaptation of the species to predicted climate change, and to develop long-term conservation strategy. The main objectives were to investigate the relationship between the functional traits leaves and C, N, P stoichiometry of Chinese fir from different geographical provenances and their relationship with the main environmental factors of provenance.

**Results:**

In this study, we measured 12 leaf functional traits on 36-year-old *Cunninghamia lanceolata* trees from 13 provenances. Analysis of variance (ANOVA) was performed to examine the variability. Redundancy analysis (RA) was computed to examine the relationship between geo-climatic factors of provenance origin and leaf functional traits while Pearson’s correlation coefficient was computed to assess inter-trait correlations. The results showed statistically significant differences (*P* < 0.01) in intraspecific leaf traits among provenances, except leaf P content. The relationships among leaf traits are consistent with the general trend observed in the leaf economic spectrum. Mean annual temperature appeared to be a key factor that influences intraspecific leaf traits variability compared to mean annual precipitation.

**Conclusion:**

These results provide useful insights about adaptation of leaf trait of Chinese fir in a changing climatic condition. Thus, our findings shed light on the importance of interspecific trait variability in Chinese fir and the potential effect of climate change.

**Supplementary Information:**

The online version contains supplementary material available at 10.1186/s12870-023-04097-y.

## Introduction

In the long process of evolution and succession, plant species with widespread geographical distribution will respond adaptively to long-term changes in climate and geographical environment, thereby forming specific geographical provenances, which also reflects the long-term adaptation strategy of plants to the growing environment [1]. Thus, the ecological importance of intraspecific variation in functional traits of a species has gained increasing attention among researchers [2–5]. It has been shown that intraspecific trait variations can provide insights about trait responses to environmental variations and genetic improvement [6, 7]. In other words, the intraspecific variation indicates the capacity of a genotype to render different phenotypic values for a trait under different environmental conditions, which is phenotypic plasticity [8]. Furthermore, intraspecific variability may explain how a given species can respond to both biotic and abiotic stresses to maintain viable populations, thereby promoting species coexistence [9]. The causes of intraspecific variation can be genetic variation, phenotypic plasticity due to differences in growing environmental conditions, or both [10]. However, it is generally opined that selection drives local adaptation, thereby resulting in genetic variation among populations growing in different environments [11].

Studies on leaf functional traits variations may provide valuable insights to assess intraspecific genetic diversity, understand the link between leaf structure and function, and the adaptive responses of a given species to environmental conditions. Such an understanding enables us to design breeding/improvement programs and strategies for genetic resources conservation. Previous studies have already confirmed negative correlation between leaf structure and leaf lifespan and leaf physiology and leaf chemical composition, such as leaf nitrogen concentration [12]. Such trade-offs between functional traits of leaf from high-light canopy positions epitomize the leaf economic spectrum, where one extreme representing an acquisitive resource strategy (e.g. low leaf dry mass per unit area and high net photosynthetic rate per unit leaf dry mass) and the other extreme representing a conservative resource strategy [12]. Leaf traits at the intraspecific level may vary among provenances, where a shift in resource use strategy can occur to adapt to the prevailing environmental conditions [13].

Plant leaves are the most important functional organs of plants. Their functional traits and differences in C, N and P stoichiometry can best reflect the adaptive strategy and competitive ability of plant to heterogeneous living environments [14]. Leaves have the largest contact area with the external environment and are most sensitive to environmental changes [15], and their traits directly affect the basic functions of plants, and reflect the adaptation strategies by plants to produce carbohydrates. They are the main organ of plants for photosynthesis and material production, which play an important role in the nutrient cycle of plants and the storage of nutrients, and has important ecological and biological evolutionary significance [16]. Plant leaf functional traits have some properties, such as relative stability, ease of measurement and rapid quantification, which can reflect the ecological adaptability of plants to various environmental factors [17]. Leaf structure and chemical contents are key factors reflecting the geographical environments of plants [18]. Despite a set of interconnected, synergistically changing functional trait combinations at the global scale, quantifying and generalizing a set of regularly changing plant resource trade-off strategies is still needed. In recent years, some scientists who studied European red pine and coffee have found that economies of inner leaves in a single plant are inconsistent with global leaf economies [19, 20]. Habitat fragmentation is one of the important factors affecting biodiversity, often resulting in population loss and fitness decline [21]. The main reason for the formation of intraspecific leaf economics spectrum is the influence of environmental factors [22]. Variations in intraspecific leaf economic traits are mainly affected by differences in habitat factors such as light, soil nutrients and water [23]. Thus, it is imperative to study the intraspecific leaf economic spectrum and its relations with seedling survival and growth in fragmented habitats to understand the adaptation and regeneration strategies of plants in fragmented habitats, as well as the maintenance of species diversity at community level.

*Cunninghamia lanceolata* (commonly known as Chinese fir) is economically valuable timber species that is used for artificial afforestation in southern China, and is widely distributed in 19 provinces ranging from 34°03’N to 20°41’N latitude [24]. It is adapted to grow in the hills with an altitude ranging from 130 to 2900 m in the entire subtropical, northern edge of the tropics, and southern edge of the warm temperate zone. The area and stock volume are about 1/5 and 1/4 of the main dominant tree species in the national plantation forests of China [25, 26]. As an ancient tree species, Chinese fir has experienced many migrations and distribution areas in the long evolutionary history. Chinese fir has also undergone adaptive changes in the long-term adaptation process of different geographical habitats and environmental conditions, and forms some unique genotypes or geographic provenances. At present, the geographical distribution of Chinese fir is not differentiated according to longitude and latitude. Instead, it shows the characteristics of "multi-center origin" and is divided into 9 provenance areas, and "three belts and five areas" [27]. There are great differences in growth characteristics such as growth rates, leaf photosynthesis, transpiration rate, seed vigor, wood density, and nutrient use efficiency among different provenances [28]. These differences may be the convergence or divergence of long-term adaptive response of Chinese fir to different growth conditions. Previous studies on Chinese fir from different geographical provenances mainly focused on evaluating differences in growth rate after introduction and cultivation, while ignoring the differences in leaf functional traits and stoichiometric characteristics between different provenances, and there was no discussion on the relationship between these traits and environmental factors.

Thus, in this study, we measured structural (leaf area, specific leaf area, leaf dry matter content, and leaf tissue density), physiological (leaf relative water content) and chemical (C, N, P concentrations) leaf traits of 36-year-old Chinese fir trees from 13 provenances grown in a common garden. The common garden approach enabled us to disentangle the latitudinal effects as well as environmental variability that affect the trait variability. The main objective of this study was to assess intraspecific trait variability of Chinese fir among different provenances that differ slightly in precipitation and temperature. The specific objectives were (1) to examine intraspecific variation at tree and provenance level; (2) to examine how leaf functional traits are related to environmental factors of the provenance origin; and (3) to examine inter-trait correlations. The analysis of the correlation between leaf functional traits and environmental factors will enable us to better understand the trade-off between adaptive characteristics and functional traits of Chinese fir for different provenances. It is also helpful to understand the reasons for the formation of the current geographical distribution pattern of Chinese fir.

## Materials and methods

### Study site

**Fig. 1:**
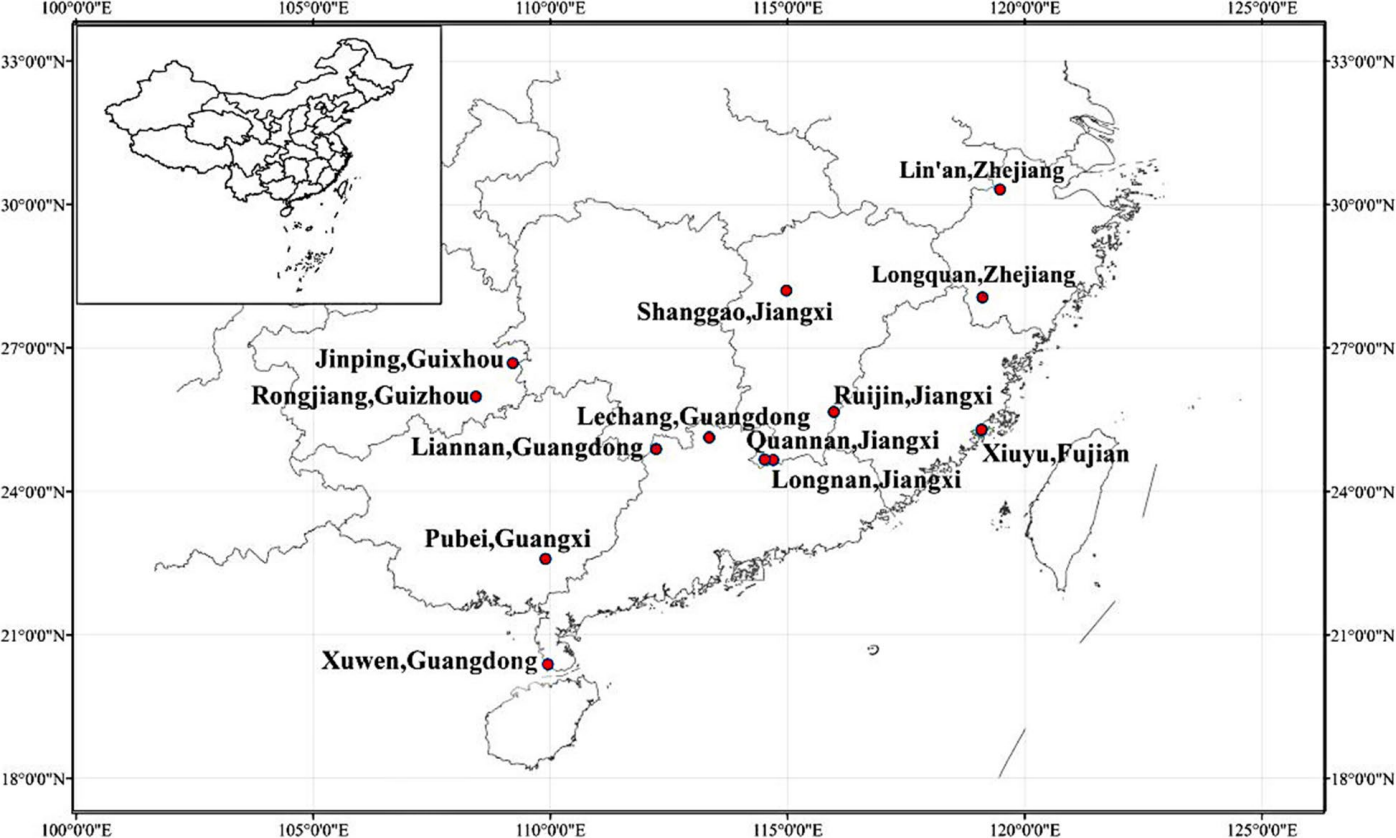
Location of provenances represented in the study

Chinese fir seeds were collected from 13 provenances (Fig. 1) in 1986, and planted with the density of 900 plants/ha in a common garden in Zhangping State-owned Forest Farm, Fujian province, located at 26°50′N and 117° 54’E. The site belongs to the central production area of Chinese fir in northern Fujian, which is characterized by mid-subtropical maritime monsoon climate. The average annual temperature is 20.3 °C, the average annual precipitation is 1508.8 mm, the frost-free period is 300 d, the relative humidity is 80%, and the sunshine hours are more than 1878 h. The soil is mainly mountain red soil. The geographic location and meteorological data of the provenances are given in Table 1. The geographic locations of the studied provenance were recorded by hand-held GPS. The meteorological data came from the Meteorological Data Center of the National Meteorological Administration of China (http://data.cma.cn/ accessed on 03 February 2022), and the meteorological data of the sampling points are the average values from 1980 to 2010.

**Table 1:** Geo-climatic conditions of different provenances usedsent study

provenance	abbreviation	Longitude	Latitude	Mean annual temperature (℃)	Mean annual precipitation(mm)	January temperature (℃)	July temperature (℃)	Growing Season Mean temperature (℃)	Growing Season precipitation(mm)	Annual Precipitation > 25 mm days(day)	Mean annual humidity(%)
Rongjiang,Guizhou	GR	108°42′E	25°98′N	18.4	1180.4	7.8	27.1	20.7	130.9	12.7	80
Jinping,Guizhou	JP	109°20′E	26°68′N	16.7	1283.0	5.5	26.7	19.2	137.5	12.4	83
Pubei,Guangxi	PB	109°89′E	22°59′N	21.7	1740.0	13.1	27.9	23.4	199.0	18.8	82
Xuwen,Guangdong	XW	109°94′E	20°38′N	23.8	1428.4	17.2	28.8	25.3	123.2	17.0	83
Liannan,Guangdong	GL	112°23′E	24°89′N	19.7	1712.0	9.2	28.6	21.9	197.6	20.1	78
Lechang,Guangdong	LC	113°35′E	25°13′N	19.9	1477.8	9.6	28.2	21.9	170.2	16.7	79
Quannan,Jiangxi	QN	114°52′E	24°67′N	18.9	1665.0	8.9	27.3	21.1	191.7	19.5	81
Longnan,Jiangxi	JL	114°70′E	24°66′N	19.2	1541.9	8.9	27.9	21.4	179.7	17.6	80
Shanggao,Jiangxi	SG	114°98′E	28°20′N	17.9	1722.6	5.7	29.3	20.6	190.6	18.6	79
Ruijin,jiangxi	JR	115°98′E	25°66′N	19.2	1652.2	8.3	28.8	21.6	192.7	19.7	79
Xiuyu,Fujian	XY	119°10′E	25°29′N	20.7	1343.4	12.3	28.5	21.7	152.2	16.1	76
Longquan,Zhejiang	LQ	119°12′E	28°06′N	18.0	1649.2	7.1	28.0	20.3	189.7	18.5	79
Lin`an,Zhejiang	LA	119°49′E	30°32′N	16.2	1463.6	3.8	28.0	18.8	158.0	15.1	78

### Measurements of leaf functional traits

For this study, we used mature trees of 36-year-old Chinese fir from 13 provenances. In April 2021, we selected three trees from each provenance and 20 healthy mature leaves grown in high light conditions on the second node of the branches from the central canopy were collected for the measurement of leaf functional traits. The following leaf functional traits were measured: leaf area (LA), specific leaf area (SLA), leaf dry matter content (LDMC), leaf tissue density (LTD) and leaf relative water content (LRWC). LA was determined by scanning the leaves with a flatbed scanner and analyzing the images using area measurement software by Image J. Leaf fresh mass and leaf saturated fresh mass were weighed. Then all leaf samples were oven-dried at 80℃ for 72 h to constant mass and weighed for their dry mass. The SLA was calculated for each leaf as the ratio of leaf area to leaf dry mass. LDMC was computed as the ratio of leaf dry mass to leaf saturated fresh mass. LTD was calculated for each leaf as the ratio of leaf dry weight to leaf volume. LRWC was calculated using the following equation [29]:

$$\mathrm{LRWC}=\frac{(\mathrm{Fresh}\;\mathrm{leaf}\;\mathrm{mass}-\mathrm{Dry}\;\mathrm{leaf}\;\mathrm{mass})}{(\mathrm{Fully}\;\mathrm{water}-\mathrm{saturated}\;\mathrm{leaf}\;\mathrm{mass}-\mathrm{Dry}\;\mathrm{leaf}\;\mathrm{mass})}\times\;100$$

A sub-sample of leaves were used for the measurement of C, N, P concentrations. All leaves from the same provenance were mixed into a single sample and grounded to pass through 0.149 mm sieve for chemical determination. Leaf C and N concentrations were analyzed using Vario Max carbon and nitrogen element analyzer (Elementar, Germany). Total leaf P concentrations were measured by a molybdate/stannous chloride method after HNO_3_-H_2_O_2_ digestion by ETHOS UP microwave digestion apparatus (Milestone, USA). All nutrient analyses were replicated three times.

### Data analysis

To examine the intraspecific variations in leaf functional traits among provenances, we conducted One-Way analysis of variance (ANOVA), and significant differences were evaluated at *P* < 0.05 level. When significant differences were observed, the Duncan post hoc test was used to conduct multiple comparisons among provenances. We also computed the coefficient of variation (CV) among provenances and individual trees within provenances. CV is a normalized measure of the degree of dispersion of the probability distribution, which reflect the absolute value of the degree of dispersion of the data. Calculation formula: CV = (SE/Mean)*100%. A larger CV represents a larger degree of dispersion of trait values [30]. It can be used to compare and measure the degree of variation between different traits [31]. All data were expressed as the mean ± standard error (SE). Redundancy analysis (RDA) was used to examine the relationships between leaf functional traits and geoclimatic conditions of the provenance origin. Finally, Pearson correlation analysis was used to examine the relationship between traits. Analysis of variance and correlation analyses were performed using SPSS 22.0 software while RDA was performed using Canoco (Version 5).

## Results

### Variations in leaf functional traits

Significant variations in leaf functional traits were observed among provenances. Specific leaf area, leaf dry matter content, leaf relative water content, and leaf tissue density showed highly significant variations among different provenances whereas leaf thickness showed significant difference among different provenances (Table 2). The coefficient of variation of the functional traits from different provenances ranged from 11.20% to 21.18%, with the largest variation was observed for specific leaf area and the smallest being for leaf dry matter content. So specific leaf area is the strongest plasticity functional trait, leaf dry matter content is the weakest. The coefficient of variation at individual tree level was relatively smaller than that of provenances. The coefficient of variation varied between 1%—18.24% for LT, 1.24%—15.01% for SLA, 0.91%—16.38% for LDMC, 0.15%—15.38% for LRWC and 3.14%—16.87% for LTD. The mean leaf thickness ranged from 0.03 to 0.04 mm; specific leaf area ranged from 58.8 to 107.6 cm2.g^–1^; leaf dry matter content ranged from 0.26 to 0.37 g.g^−1^; leaf relative water content ranged from Chinese fir trees from 0.53% to 0.93; and leaf tissue density ranged from 0.29 to 0.41 g.(cm^3^)^−1^. Chinese fir trees from LC had the smallest specific leaf area, but had the largest leaf dry matter content, and the largest leaf relative water content. Chinese fir trees from LQ had the largest specific leaf area. The leaf tissue density of Chinese fir trees from XW was the largest whereas the leaf relative water content of Chinese fir trees from XY was the smallest. The leaf dry matter content of Chinese fir trees from GR was the smallest.

**Table 2:** Structural and physiological leaf traits of *Cunninghamia lanceolata* from different provenances (Mean ± SE). Means followed by different lower case letters are significantly different among provenances. Values in the parenthesis are coefficient of variation (CV)

Provenance	LT/mm	SLA/cm^2^.g^−1^	LDMC/g.g^−1^	LRWC/%	LTD/g.(cm^3^)^−1^
GR	0.04 ± 0.00ab	92.23 ± 4.25de	0.26 ± 0.04ab	0.77 ± 0.08b	0.31 ± 0.02ab
	(8.83%)	(4.61%)	(16.38%)	(10.63%)	(6.82%)
JP	0.03 ± 0.00a	92.60 ± 4.93de	0.31 ± 0.01ab	0.83 ± 0.12bc	0.33 ± 0.03abc
	(12.66%)	(5.32%)	(4.56%)	(14.46%)	(10.20%)
PB	0.04 ± 0.00abc	89.19 ± 1.11d	0.29 ± 0.00ab	0.77 ± 0.03b	0.29 ± 0.02a
	(6.99%)	(1.24%)	(0.91%)	(4.35%)	(6.30%)
XW	0.04 ± 0.00abc	79.86 ± 11.47bcd	0.30 ± 0.01ab	0.73 ± 0.07b	0.35 ± 0.04abcd
	(9.82%)	(14.37%)	(4.62%)	(9.45%)	(10.89%)
GL	0.04 ± 0.01bc	63.06 ± 6.66ab	0.33 ± 0.01bc	0.72 ± 0.01b	0.37 ± 0.04bcd
	(18.24%)	(10.56%)	(2.17%)	(1.24%)	(9.87%)
LC	0.04 ± 0.00abc	58.80 ± 6.39a	0.37 ± 0.03c	0.93 ± 0.02c	0.39 ± 0.01 cd
	(11.10%)	(10.86%)	(7.26%)	(2.65%)	(3.14%)
QN	0.04 ± 0.00abc	87.25 ± 6.66d	0.29 ± 0.00ab	0.73 ± 0.06b	0.32 ± 0.01abc
	(4.23%)	(7.64%)	(0.99%)	(8.87%)	(3.92%)
LN	0.04 ± 0.00c	67.52 ± 4.58abc	0.31 ± 0.02ab	0.71 ± 0.02b	0.30 ± 0.06a
	(7.87%)	(6.78%)	(7.99%)	(3.46%)	(18.87%)
SG	0.03 ± 0.00a	65.19 ± 9.78ab	0.33 ± 0.03bc	0.53 ± 0.03a	0.41 ± 0.05d
	(8.14%)	(15.01%)	(9.86%)	(5.04%)	(12.72%)
JR	0.04 ± 0.00c	78.57 ± 9.38bcd	0.30 ± 0.02ab	0.83 ± 0.00b	0.29 ± 0.03a
	(1.00%)	(11.94%)	(8.13%)	(0.15%)	(11.42%)
XY	0.03 ± 0.00ab	89.04 ± 2.38d	0.27 ± 0.03a	0.56 ± 0.09a	0.33 ± 0.02abc
	(7.58%)	(2.68%)	(10.72%)	(15.37%)	(4.94%)
LQ	0.03 ± 0.00a	107.56 ± 13.12e	0.31 ± 0.00ab	0.81 ± 0.04bc	0.29 ± 0.03a
	(10.44%)	(12.20%)	(1.32%)	(5.24%)	(11.56%)
LA	0.04 ± 0.00abc	82.68 ± 6.11bc	0.29 ± 0.00ab	0.82 ± 0.10bc	0.30 ± 0.03a
	(3.30%)	(7.39%)	(1.31%)	(12.57%)	(9.47%)
Overall mean & CV	0.04 ± 0.01	81.04 ± 15.49	0.30 ± 0.03	0.75 ± 0.12	0.33 ± 0.05
	(15.02%)	(19.11%)	(11.20%)	(16.58%)	(15.75%)

*LT*  leaf thickness, *SLA*  Specific leaf area, *LDMC*   Leaf dry matter content, *LRWC *  Leaf relative water content, *LTD*  Leaf tissue density

Similarly highly significant variations in chemical traits were observed among provenances (Table 3). While the N content varied significantly among provenances, the P content did not varied significantly. The coefficient of variation of the chemical traits from different provenances ranged from 10.33% to 23.24%, with the largest variation was observed for C:N and N:P ratios and the smallest being for C and P contents. So C:N and N:P are the strongest plasticity functional trait, C and P contents are the weakest chemical traits. At individual tree level, the coefficient of variation ranged from 0.65% to 14.03% for C content, 4.02% to 17.55% for N content, 4.24% to 18.20% for C:N ratio, 0.25% to 8.61% for C:P ratio, and 1.27% to 8.61% for N:P ratio. The mean C content ranged from 357.5 to 499.32 g/kg^−1^; The N content ranged from 18.80 to 28.97 g/kg^−1^; the P content ranged from 0.58 to 0.68 g/kg^−1^; C:N ratio ranged 13.38 to 26.20; C:P ratio ranged from 535.87 to V; and N:P ratio ranged from 27.61 to 58.93 (Table 3). Chinese fir trees from LC had the highest leaf C content, but the lowest P content. Chinese fir trees from LQ had the lowest leaf C:P ratio while those from Jiangxi had the smallest leaf N:P ratio. Chinese fir trees from Zhejiang Lin'an had the lowest C content and the smallest C:N ratio. The leaf N content of Chinese fir trees from JP was the largest. Chinese fir trees from JR had the smallest N content, but the largest C:N and C:P ratios. N:P ratio was the largest for Chinese fir trees from GR.

**Table 3:** C, N and P contents and their stoichiometry of *Cunninghamia lanceolata* from different provenances (Mean ± SE). Means followed by different lower case letters are significantly different among provenances. Values in the parenthesis are coefficient of variation (CV)

Provenance	C/g.kg^−1^	N/g.kg^−1^	P/g.kg^−1^	C:N	C:P	N:P
GR	407.98 ± 2.43bc	26.34 ± 3.67bcd	0.63 ± 0.06a	15.84 ± 2.5ab	655.03 ± 56.41bcd	48.56 ± 0.61e
	(0.60%)	(13.95%)	(9.06%)	(15.77%)	(8.61%)	(1.27%)
JP	466.42 ± 19.59def	28.97 ± 2.71d	0.63 ± 0.06a	16.31 ± 2.25abc	732.36 ± 25.32ef	41.03 ± 1.51cde
	(4.20%)	(9.36%)	(9.26%)	(13.78%)	(3.46%)	(3.69%)
PB	452.2 ± 2.92cde	20.39 ± 0.82abc	0.69 ± 0.07a	22.22 ± 0.94def	615.48 ± 26.89bc	29.85 ± 3.92a
	(0.65%)	(4.02%)	(9.92%)	(4.24%)	(4.37%)	(13.13%)
XW	474.59 ± 4.82ef	23.23 ± 2.48abcd	0.59 ± 0.00a	20.65 ± 2.03bcde	807.68 ± 1.98 g	39.27 ± 4.1bcd
	(1.02%)	(10.70%)	(0.26%)	(9.84%)	(0.25%)	(10.44%)
GL	387.71 ± 9.90ab	25.56 ± 3.78abcd	0.61 ± 0.03a	15.58 ± 2.78ab	638.12 ± 20.15bcd	42.14 ± 6.83de
	(2.55%)	(14.78%)	(5.29%)	(17.85%)	(3.16%)	(16.20%)
LC	499.32 ± 13.79f	20.76 ± 2.46abc	0.62 ± 0.13a	24.41 ± 3.14ef	784.35 ± 20.71 fg	32.14 ± 3.85ab
	(2.76%)	(11.85%)	(2.16%)	(12.86%)	(2.64%)	(11.96%)
QN	427.26 ± 59.92bcd	25.29 ± 2.24abcd	0.60 ± 0.02a	16.84 ± 1.03ab	653.79 ± 4.96bcd	42.17 ± 2.93de
	(14.03%)	(8.86%)	(2.53%)	(6.14%)	(0.76%)	(6.94%)
JL	424.82 ± 18.6bcd	19.7 ± 2.21ab	0.71 ± 0.05a	21.72 ± 1.4cdef	597.95 ± 14.04b	27.61 ± 1.28a
	(4.38%)	(11.22%)	(6.53%)	(6.46%)	(2.35%)	(4.62%)
SG	415.86 ± 12.96bc	22.98 ± 2.79abcd	0.64 ± 0.05a	18.34 ± 2.14abcd	617.5 ± 30.79bc	36.08 ± 5.73abcd
	(3.12%)	(12.13%)	(7.58%)	(11.68%)	(4.99%)	(15.88%)
JR	486.27 ± 12.07ef	18.80 ± 2.04a	0.58 ± 0.02a	26.20 ± 3.14f	811.1 ± 2.56 g	35.26 ± 1.35abcd
	(2.48%)	(10.87%)	(3.31%)	(11.97%)	(0.32%)	(3.82%)
XY	442.1 ± 8.44cde	23.37 ± 4.10abcd	0.62 ± 0.07a	19.54 ± 3.56bcde	674.36 ± 40.64 cd	32.76 ± 0.87abc
	(1.91%)	(17.55%)	(11.90%)	(18.20%)	(6.03%)	(2.65%)
LQ	364.62 ± 8.49a	22.46 ± 2.83abcd	0.68 ± 0.02a	16.50 ± 2.11abc	535.87 ± 17.20a	32.91 ± 3.39abc
	(2.33%)	(12.61%)	(2.57%)	(12.77%)	(3.21%)	(10.29%)
LA	357.5 ± 8.13a	27.52 ± 4.78 cd	0.58 ± 0.11a	13.38 ± 2.29a	695.98 ± 11.25de	58.93 ± 3.70f
	(2.27%)	(17.36%)	(18.23%)	(17.15%)	(1.62%)	(6.29%)
Overall mean & CV	431.28 ± 47.32	23.49 ± 4.24	0.63 ± 0.07	19.04 ± 4.36	678.43 ± 85.33	38.36 ± 8.92
	(10.97%)	(18.07%)	(10.33%)	(22.92%)	(12.58%)	(23.24%)
*P*-value	0.000**	0.050*	0.244	0.000**	0.000**	0.000**

* indicates a significant relationship (*P*-value < 0.05); ** indicates a very significant relationship (*P*-value < 0.01)

### Relationship between geoclimatic conditions of provenances and leaf functional traits

**Fig. 2:**
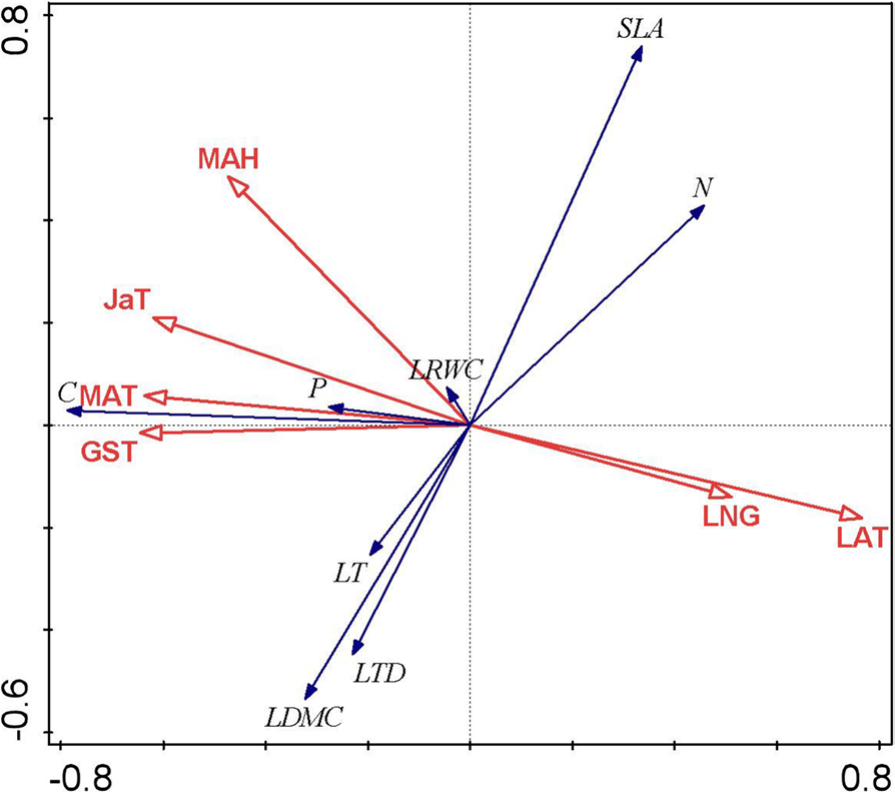
Redundancy analysis functional traits of *C. lanceolata* mature forests from different provenances. Where LAT, GST, MAT, JaT, LNG, MAH stand for Latitude, growing season mean temperature, mean annual temperature, January temperature, Longitude, mean annual humidity. SLA, LTD, LT, LDMC, and LRWC represent specific leaf area, leaf tissue density, leaf thickness, leaf dry matter content, and leaf relative water content, respective

Redundancy Analysis was performed to examine the relationship between geoclimatic conditions of provenances and leaf functional traits (Fig. 2). The results showed that LRWC was significantly positively correlated with MAH, and significantly negatively correlated with longitude and latitude. Leaf C and P contents were significantly positively correlated with MAT, GST and JaT, and significantly negatively correlated with longitude and latitude. Additional PAC analysis and cluster analysis of Non-metric multidimensional scaling (NMDS) were used to display the functional traits vary across the provenances. The PCA analysis indicated that the first two axes explained 50.3% of differences between functional traits of different geographical provenances, with PC1 and PC2 explaining 30.2% and 20.1% (Fig. S3). The principal component values indicated the SLA and LDMC was the main characteristic value to explain the changes of leaf functional traits and stoichiometry among provenances. The NMDS analysis showed all provenances were grouped into 4 groups, in which LA and LC were grouped separately, LC, XW, JR and JP were grouped together, and the other 7 provenances were grouped together (Fig. S4).

### Correlations among leaf functional traits

Correlation analyses revealed that leaf thickness was significantly negatively correlated with specific leaf area, leaf tissue density, and leaf N content, and was significantly positive correlated with C:N ratio (Table 4). There was a significant negative correlation between leaf tissue density and leaf dry matter content. There was a highly significant positive correlation between leaf dry matter content and leaf tissue density. Significant positive correlation was observed between leaf relative water content and C:P ratio. Leaf C content was significantly positively correlated with C:P and C:N, and was significantly negatively correlated with N:P content. Leaf N content was highly significantly negative correlation with P content, and was highly significant positive correlation with N:P ratio. There was a very significant negative correlation between P content and C:P ratio while significantly negative correlation was observed between C:N ratio and N:P ratio.

**Table 4:** Correlation coefficients of functional traits of *Cunninghamia lanceolata* from different provenances.*represent the significant relation at the 0.05 level, **represent the significant relation at the 0.01 level

	LT	SLA	LDMC	LRWC	LTD	C	N	P	C:N	C:P
SLA	-0.458**									
LDMC	0.151	-0.591**								
LRWC	0.220	0.080	0.146							
LTD	-0.332*	-0.524**	0.434**	-0.249						
C	0.032	-0.201	0.207	0.200	0.103					
N	-0.342*	0.216	-0.102	0.071	0.060	-0.230				
P	0.109	0.028	0.144	-0.144	-0.193	0.009	-0.273			
C:N	0.332*	-0.291	0.168	0.079	0.001	0.653**	-0.862**	0.151		
C:P	0.126	-0.246	0.128	0.344*	0.141	0.600**	0.036	-0.543**	0.311	
N:P	-0.148	0.137	-0.277	0.113	0.020	-0.377*	0.690**	-0.405*	-0.678**	0.157

*LT*  leaf thickness, *SLA*  Specific leaf area, *LDMC*   Leaf dry matter content, *LRWC*  Leaf relative water content, *LTD*  Leaf tissue densi

## Discussion

Tree after long-term growth in different ecological environments undergo adaptive geographic variation to the local habitat, forming different geographic provenances [32]. However, each species has a certain range of adaptation to the environment, and the variation of functional traits has a certain limit too [33]. The results from the present study demonstrate high intraspecific variability in leaf traits among provenances and individual trees within provenances. The high intraspecific variability represents strong phenotypic plasticity of plant, and it is the major means of plants cope with environmental heterogeneity [34]. At the provenance level, the variation in intraspecific leaf functional traits did not strictly follow the precipitation gradient, but tended to follow variation in mean annual temperature of the provenance origin. Generally, intraspecific trait variability at the provenance level is shaped by edaphic-climatic factors and genetic effect [3, 4, 35]. In our study, LDMC showed low variability while SLA showed high variability at the provenance level. These traits are strongly correlated with plant growth rate and resource use strategies [36–38]. Plants with smaller leaf area have strong ability to maintain own water and nutrients, and can better adapt to drought and resource-poor environments [39, 40]. For instance, high SLA but low LDMC predict the ability of plant leaves to capture light resources, thereby optimizing photosynthetic efficiency under low light availability [41–44]. Plants growing in relatively dry environment tend to have high LMA, which might offer an adaptation to shield the leaf from desiccation [45]. LMA also epitomizes the connection between leaf structure and function [12], and hence is the most studied trait. It has been shown that LDMC is extremely variable under different soil moisture conditions [46]. This, in turn, is associated with root growth differences at provenance and individual tree levels [47]; suggesting that restricted root growth is detrimental in drought-prone areas. In our study, LRWC showed relatively moderate variation among provenances, which depicts differences in water use efficiency, which in turn are associates with improved gas exchange efficiency [2]. It should be noted that LRWC and drought stress are positively correlation, and if the leaf relative water content increases, the drought stress of plant will increase.

Similarly, large variability among chemical traits was observed for leaf N content, C:N and N:P ratios. Variation in leaf chemical traits can be related to differences in soil nutrient availability, nutrient use efficiency or the extent of root growth and development among trees [6, 48]. In our study, there was no significant difference in leaf P content among provenances. This is expected as the entire habitat of Chinese fir is deficient in plant available P [49]. The N:P ratio reflects the restriction of N and P on plant growth and development [50]. If N:P ratio is more than 16, the plant would have faced P stressed [51]. In our experiment, the N:P ratio of Chinese fir from 13 different provenances studied was more than 16. This shows that plant available P is deficient in various source habitat conditions. In China, the leaf P content of plants is generally lower than the global level, which may be due to low plant available soil P content [52]. However, we observed as high as 18% variation in leaf P content among individual trees. This could be attributed to individual tree’s adaptation mechanism to low soil P availability, including root architectural changes, root secretion that chelates insoluble P [53, 54], maintaining low P requirements in the tissue and redistribution of P from old and senescent to more active tissues [55]. The C:N and N:P ratios are related with root development for increased acquisition of soil nutrients and better light capture that drives photosynthetic rate. It has been shown that the C: N ratio of leaves reflect the utilization efficiency of N [56, 57], while N:P ratio reflects N and P limitations on plant growth [58].

Among environmental factors of the provenance origin, Latitude, growing season mean temperature, mean annual temperature, January temperature, Longitude and mean annual humidity had a great influence on the functional traits of Chinese fir. Mean annual precipitation and growing season precipitation did not have an influence on functional traits. Despite weak precipitation gradient across all provenances studied, there were significant positive correlations between the mean annual humidity and LRWC. Chinese fir trees from Lechnag, Guangdong had the smallest SLA, the largest LDMC, the largest LRWC, the highest leaf C content, and the lowest P content. This provenance is characterized by good growing season precipitation and temperature. Similarly, Chinese fir trees from XW, which is characterized by high mean annual temperature and January temperature, had high leaf C content. These good climatic conditions favor high photosynthetic rate that in turn resulted in large leaf mass investment. SLA was high for Chinese fir trees from LQ, JP and GR, where January temperature is sub-optimal. Thus, high SLA offers an adaptation to capture more light resources to optimize photosynthetic rate under low temperature conditions and availability of light [41–44]. Our results show that mean annual precipitation does not affect the intraspecific trait variability in the way same as changes in mean annual temperature. Therefore, it seems that water consumption traits, such as LRWC are controlled by an increase in temperature because of stomatal control, despite increases in precipitation. Similar results have been reported for *P. pallida* where temperature is a key factor that governs intraspecific variability in water consumption traits [2].

We also found a negative correlation between latitude and C, P content. Latitude is often related with degree of insolation and affects temperature. In our case, temperature and latitude had opposite effects on functional traits due to the fact that the studied provenances belong to the southern warm regions. Therefore, altitude has a greater effect on temperature than latitude. Generally trees exposed to low light conditions tend to have larger SLA and high leaf N content for increased synthesis of light-capturing proteins, such as chlorophyll under low intensity of light [59]. Indeed, the low leaf C content is the results of reduced photosynthetic rate due to low insolation at higher latitude.

Our results also showed SLA and LDMC are important leaf traits, which describe leaf mass investment and leaf structure and are related to leaf lifespan, chemical composition of leaf, and gas exchange rate of leaf [12]. Similar results with LDMC have been reported for *Prosopis pallida* and other plant species [2, 13, 60]. The high leaf N content, which also showed negative correlation with leaf thickness (LT) suggest that Chinese fir could be have adaptation to resource acquisition with quick returns from leaf investments.

Global climate change generally predicted to result in increased temperature, drought and reduced frequency of rainfall in the southern hemisphere, including China [61]. Although our results did not suggest unfavorable effect of increased temperature on Chinese fir provenances, the decrease in precipitation might have an effect on growth of Chinese fir trees. Under the predicted climate change scenario, Chinese fir provenances would be subjected to water stress that in turn considerably affects growth and development of the species. Therefore, the successful establishment of Chinese fir forests in the future largely depends on the intensity and frequency of these predicted climatic events. Thus, selection of drought-tolerant phenotypes would be a good strategy to cope with the changing climatic conditions in future planting of Chinese fir as moderate genetic variability in intraspecific leaf traits was observed among Chinese fir provenances.

## Conclusions

Leaf functional traits of Chinese fir provenances showed high intraspecific variability. Both leaf structural and chemical traits shows considerable variations at provenance and individual tree levels. The relationships among leaf traits are consistent with the general trend observed in the leaf economic spectrum, with one end showing resource acquisition strategy (high leaf N content and low LT and the other end showing opposite traits that represent a conservative resource strategy. Mean annual temperature appeared to be a key factor that influences intraspecific leaf traits variability compared to mean annual precipitation. These results provide useful insights about adaptation of leaf trait of Chinese fir in a changing climatic condition.

## Supplementary Information

**Additional file 1: Table S1.** Leaf traits of *Cunninghamia lanceolata* from different provenances.

**Additional file 2: Figure S1.** Structural and physiological leaf traits of *Cunninghamia lanceolata* from different provenances. Means followed by different lower case letters are significantly different among provenances. **Figure S2.** C, N and P contents and their stoichiometry of *Cunninghamia lanceolata* from different provenances. **Figure S3. **Principal component analysis of functional traits of C. *lanceolata* mature forests from different provenances. **Figure S4.** Non-metric multidimensional scaling of C. *lanceolata* mature forests from different provenances. **Figure S5.** Sample Collection in common garden.

## Data Availability

The data set used in this study can be made available from the corresponding author on reasonable request.
