# Supplementary material for: Intraspecific variations in leaf functional traits of Cunninghamia lanceolata provenances
Source: BMC Plant Biol. 2023 Feb 13;23:92. doi: 10.1186/s12870-023-04097-y (PMC9926855; doi:10.1186/s12870-023-04097-y)
Supplement: Supplementary file 1 — Additional file 1: Table S1. Leaf traits of Cunninghamia lanceolata from different provenances. [file 12870_2023_4097_MOESM1_ESM.docx]

**Table S1**. Leaf traits of *Cunninghamia lanceolata* from different provenances (Mean ± SE).

| provenance | Leaf thickness(mm) | Leaf area(cm^2^) | Leaf fresh mass(g) | Leaf  dry mass(g) | Leaf saturated fresh mass(g) |
| --- | --- | --- | --- | --- | --- |
| Longquan,Zhejiang | 0.03±0.00 | 17.79±2.74 | 0.46±0.01 | 0.16±0.01 | 0.53±0.01 |
|  | 10.44% | 15.38% | 1.36% | 3.25% | 1.93% |
| Jinping,Guizhou | 0.03±0.00 | 28.49±1.88 | 0.88±0.07 | 0.31±0.02 | 1.00±0.05 |
|  | 12.66% | 6.61% | 8.24% | 4.96% | 4.66% |
| Xiuyu,Fujian | 0.03±0.00 | 25.69±2.42 | 0.64±0.12 | 0.29±0.03 | 1.05±0.02 |
|  | 7.58% | 9.41% | 18.45% | 11.16% | 1.94% |
| Xuwen,Guangdong | 0.04±0.00 | 17.37±4.16 | 0.72±0.07 | 0.22±0.05 | 0.9±0.07 |
|  | 9.82% | 23.97% | 9.36% | 21.15% | 7.31% |
| Shanggao,Jiangxi | 0.03±0.00 | 15.33±2.02 | 0.49±0.04 | 0.24±0.01 | 0.72±0.04 |
|  | 8.14% | 13.16% | 7.72% | 5.23% | 6.15% |
| Lin`an,Zhejiang | 0.04±0.00 | 24.49±0.89 | 0.90±0.04 | 0.30±0.03 | 1.04±0.1 |
|  | 3.30% | 3.63% | 4.70% | 10.51% | 9.78% |
| Lechang,Guangdong | 0.04±0.00 | 13.95±0.14 | 0.49±0.26 | 0.24±0.03 | 0.67±0.04 |
|  | 11.10% | 0.99% | 52.51% | 10.86% | 6.08% |
| Longnan,Jiangxi | 0.04±0.00 | 19.00±3.05 | 0.64±0.02 | 0.24±0.03 | 0.80±0.04 |
|  | 7.87% | 16.04% | 3.51% | 12.67% | 4.53% |
| Liannan,Guangdong | 0.04±0.01 | 21.64±2.49 | 0.85±0.17 | 0.35±0.07 | 1.04±0.20 |
|  | 18.24% | 11.49% | 19.58% | 20.96% | 19.40% |
| Quannan,Jiangxi | 0.04±0.00 | 21.76±3.88 | 0.68±0.09 | 0.25±0.03 | 0.85±0.10 |
|  | 4.23% | 17.84% | 13.53% | 11.16% | 11.57% |
| Pubei,Guangxi | 0.04±0.00 | 19.52±0.29 | 0.61±0.15 | 0.22±0.00 | 0.79±0.14 |
|  | 6.99% | 1.47% | 24.65% | 0.33% | 18.03% |
| Rongjiang,Guizhou | 0.04±0.00 | 22.30±2.82 | 0.77±0.03 | 0.24±0.04 | 0.85±0.12 |
|  | 8.83% | 12.66% | 3.31% | 15.66% | 14.67% |
| Ruijin,jiangxi | 0.04±0.00 | 19.85±2.44 | 0.80±0.01 | 0.26±0.04 | 0.91±0.01 |
|  | 1.00% | 12.29% | 1.50% | 13.89% | 0.68% |
| Overall mean & CV | 0.04±0.01 | 20.55±4.70 | 0.7±0.16 | 0.26±0.06 | 0.86±0.18 |
|  | 15.02% | 22.86% | 22.50% | 22.18% | 20.71% |

Means followed by different lower case letters are significantly different among provenances; Values in the parenthesis are coefficient of variation (CV)
