# Supplementary material for: Intraspecific variations in leaf functional traits of Cunninghamia lanceolata provenances
Source: BMC Plant Biol. 2023 Feb 13;23:92. doi: 10.1186/s12870-023-04097-y (PMC9926855; doi:10.1186/s12870-023-04097-y)
Supplement: Supplementary file 2 — Additional file 2: Figure S1. Structural and physiological leaf traits of Cunninghamia lanceolata from different provenances. Means followed by different lower case letters are significantly different among provenances. Figure S2. C, N and P contents and their stoichiometry of Cunninghamia lanceolata from different provenances. Figure S3. Principal component analysis of functional traits of C. lanceolata mature forests from different provenances. Figure S4. Non-metric multidimensional scaling of C. lanceolata mature forests from different provenances. Figure S5. Sample Collection in common garden. [file 12870_2023_4097_MOESM2_ESM.docx]

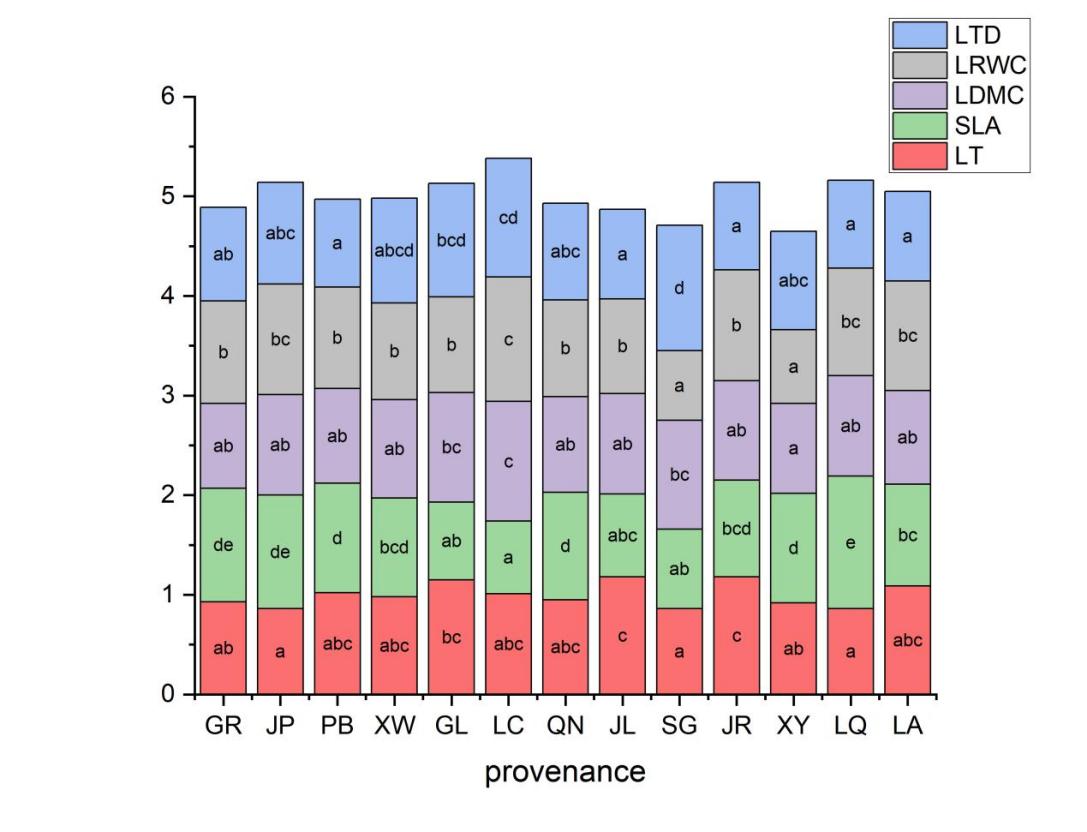


**Figure S1.** Structural and physiological leaf traits of *Cunninghamia lanceolata* from different provenances. Means followed by different lower case letters are significantly different among provenances. Values in the parenthesis are coefficient of variation (CV).

Note: Due to the differences in the value of different functional traits, we rescaled the measured values of functional traits. It is the ratio of the value to the mean value of the functional trait.


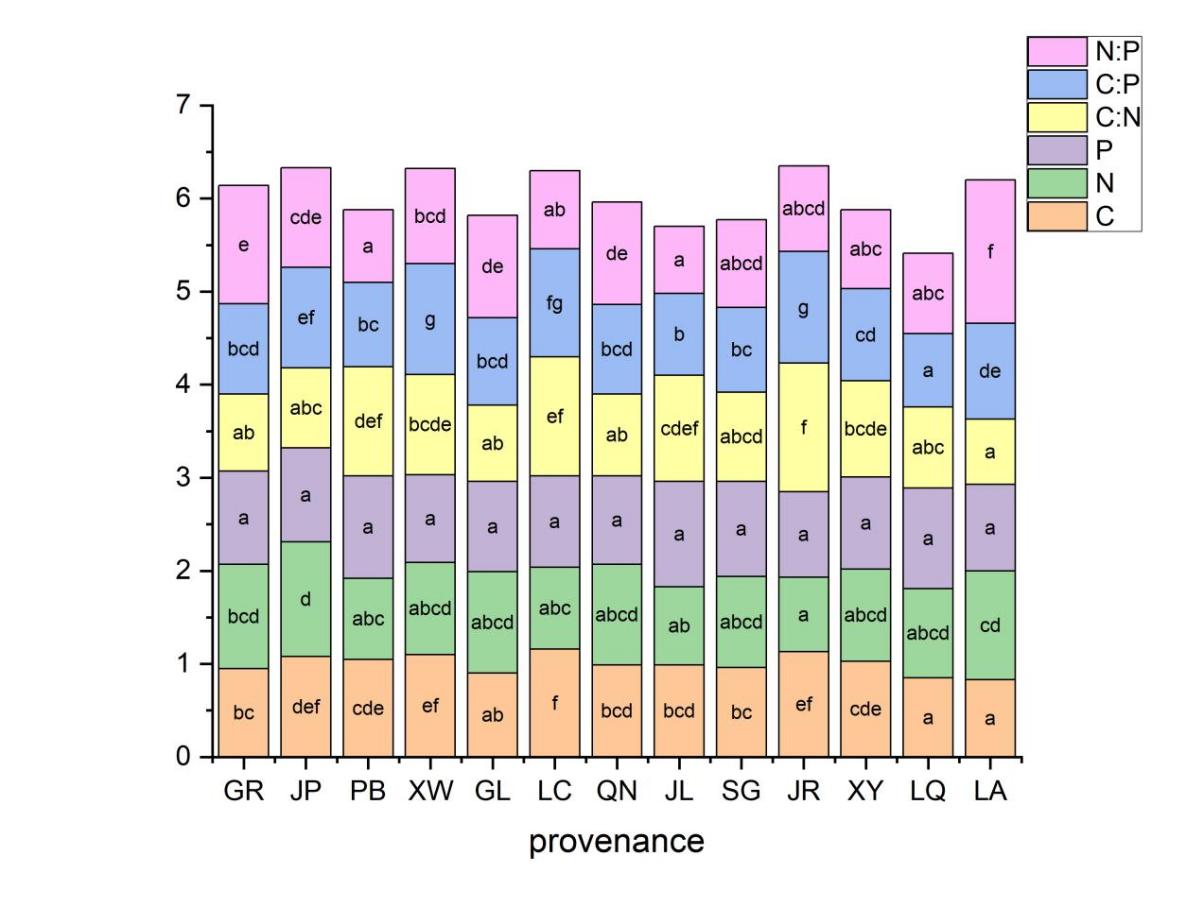


**Figure S2.** C, N and P contents and their stoichiometry of *Cunninghamia lanceolata* from different provenances. Means followed by different lower case letters are significantly different among provenances. Values in the parenthesis are coefficient of variation (CV).


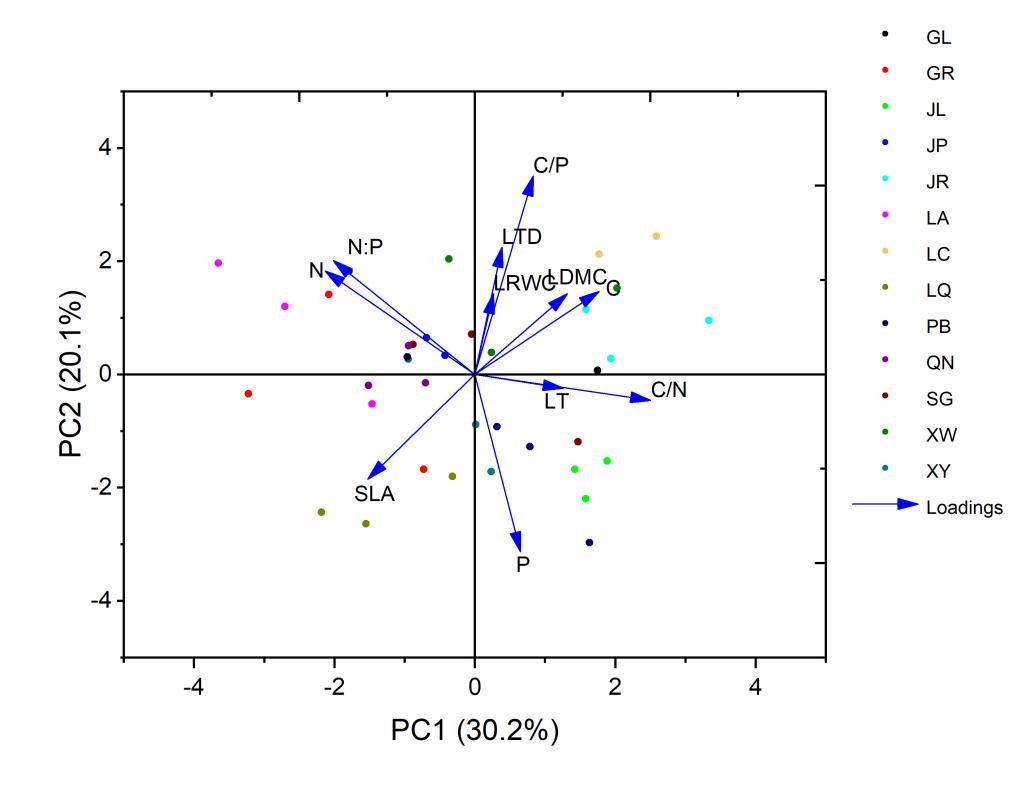


**Figure S3.** Principal component analysis of functional traits of C. *lanceolata* mature forests from different provenances.

LT = leaf thickness; SLA = Specific leaf area; LDMC = Leaf dry matter content; LRWC = Leaf relative water content; LTD = Leaf tissue density.


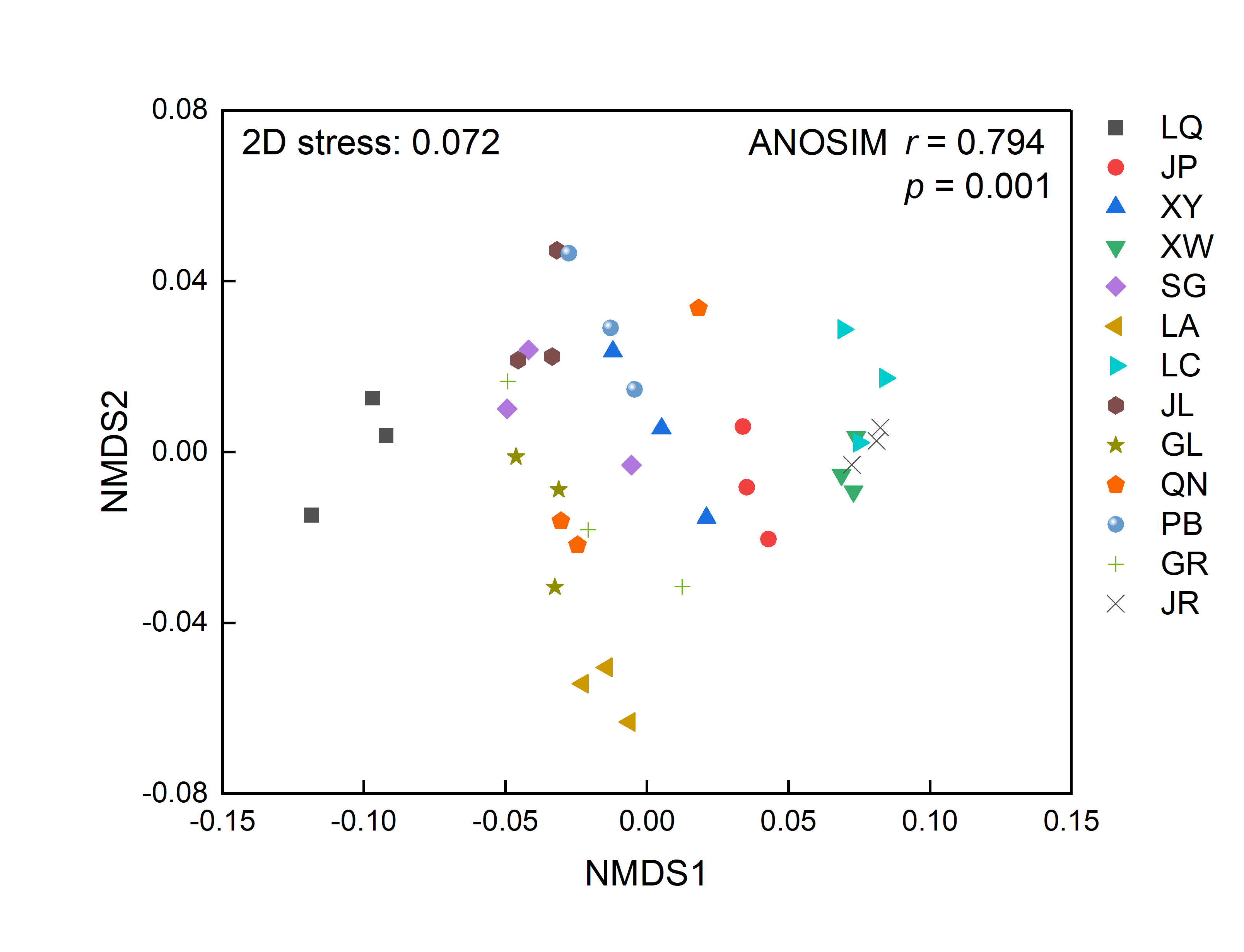


# Figure S4. Non-metric multidimensional scaling of C. *lanceolata* mature forests from different provenances.


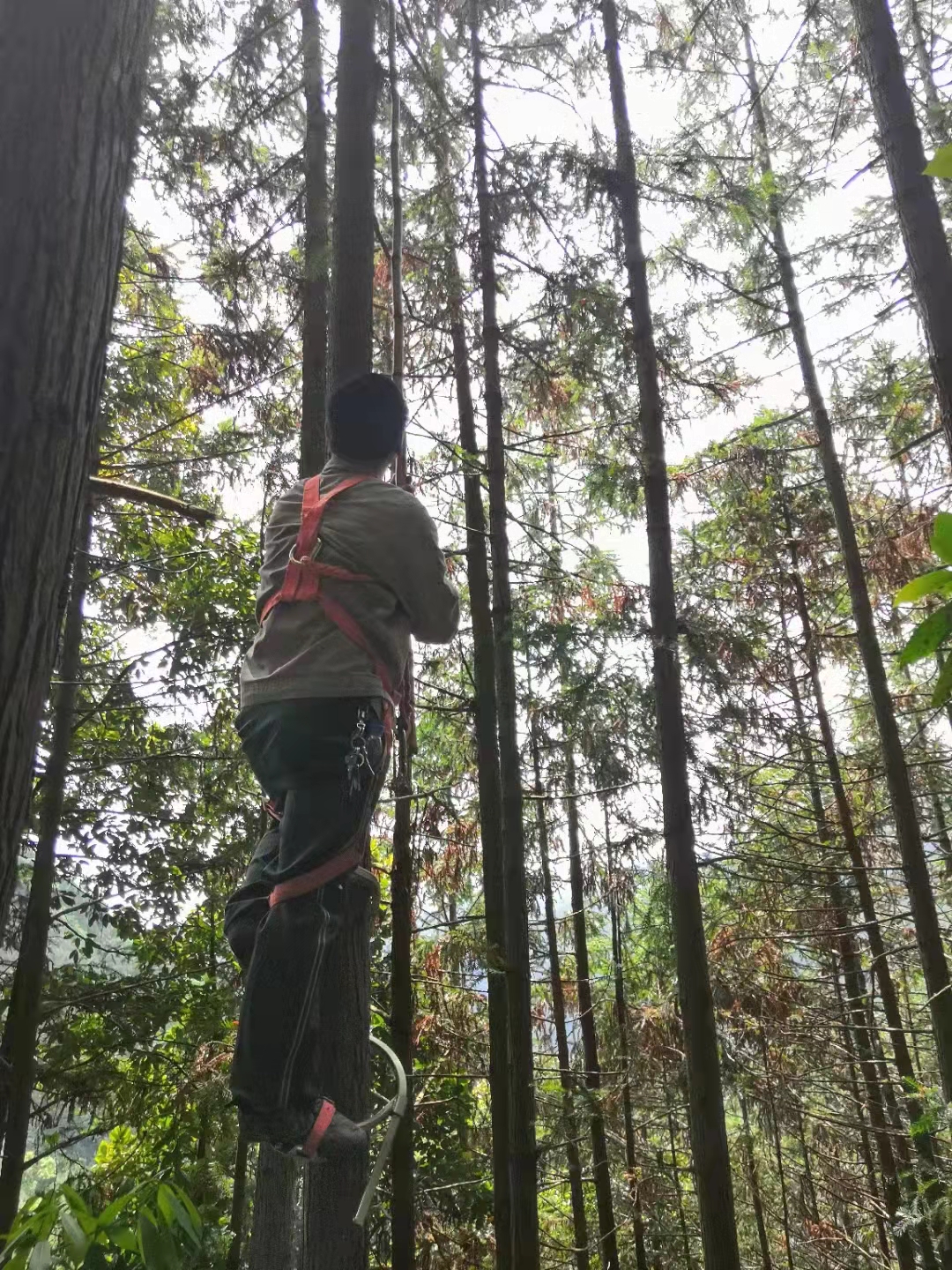


**Figure S5. Sample Collection in common garden**
